# Correction: Su et al. NADPH Oxidase Subunit CYBB Confers Chemotherapy and Ferroptosis Resistance in Mesenchymal Glioblastoma via Nrf2/SOD2 Modulation. *Int. J. Mol. Sci.* 2023, *24*, 7706

**DOI:** 10.3390/ijms26104837

**Published:** 2025-05-19

**Authors:** I-Chang Su, Yu-Kai Su, Syahru Agung Setiawan, Vijesh Kumar Yadav, Iat-Hang Fong, Chi-Tai Yeh, Chien-Min Lin, Heng-Wei Liu

**Affiliations:** 1 Graduate Institute of Clinical Medicine, College of Medicine, Taipei Medical University, Taipei City 11031, Taiwan; ichangsu@gmail.com (I.-C.S.); yukai.su@gmail.com (Y.-K.S.); 18149@s.tmu.edu.tw (I.-H.F.); 2 Department of Neurology, School of Medicine, College of Medicine, Taipei Medical University, Taipei City 11031, Taiwan; 3 Division of Neurosurgery, Department of Surgery, Taipei Medical University-Shuang Ho Hospital, New Taipei City 23561, Taiwan; 4 Taipei Neuroscience Institute, Taipei Medical University, Taipei City 11031, Taiwan; 5 International Ph.D. Program in Medicine, College of Medicine, Taipei Medical University, Taipei City 11031, Taiwan; setiawan.syahru@gmail.com; 6 Department of Medical Research & Education, Taipei Medical University-Shuang Ho Hospital, New Taipei City 23561, Taiwan; vijeshp2@gmail.com (V.K.Y.); ctyeh@s.tmu.edu.tw (C.-T.Y.); 7 Continuing Education Program of Food Biotechnology Applications, College of Science and Engineering, National Taitung University, Taitung 95092, Taiwan

In the original publication [1], there was a mistake in the Institutional Review Board Statement section of this article. The authors want to add the IRB number and Joint Biobank statement to the Institutional Review Board Statement section of this article. The authors state that the scientific conclusions are unaffected. This correction was approved by the Academic Editor. The original publication has also been updated. The IRB approval number and Joint Biobank statement should be corrected as follows.

**Institutional Review Board Statement:** The study was approved by the Joint Institutional Review Board (JIRB) of the Taipei Medical University–Shuang Ho Hospital (Approval no.: N202101069, NBCT No.200039, NBCT No.220104, N202101065, N201801070, N202002052 and N202008022). Tissue samples from patients with primary and recurrent GBM were obtained from the Taipei Medical University—Joint Biobank, and complied with the recommendations of the Declaration of Helsinki for Biomedical Research.
