# Supplementary material for: NADPH Oxidase Subunit CYBB Confers Chemotherapy and Ferroptosis Resistance in Mesenchymal Glioblastoma via Nrf2/SOD2 Modulation
Source: Int J Mol Sci. 2023 Apr 22;24(9):7706. doi: 10.3390/ijms24097706 (PMC10178261; doi:10.3390/ijms24097706)
Supplement: Supplementary file 1 [file ijms-24-07706-s001.zip › ijms-2260301-supplementary.pdf]

Supplementary Figures

NADPH Oxidase Subunit CYBB Confers Chemoresistance and Ferroptosis Vulnerability in Mesenchymal Glioblastoma via Nrf2/SOD2 Modulation

I-Chang Su[1,2,3,4,†], Yu-Kai Su [1,2,3,4,†], Syahru Agung Setiawan [5,6], Vijesh Kumar Yadav[6], Iat-Hang Fong[1,2,3,4], Chi-Tai Yeh [6,7], Chien-Min Lin [1,2,3,4*], Heng-Wei Liu [1,2,3,4*]

1 Graduate Institute of Clinical Medicine, College of Medicine, Taipei Medical University, Taipei City 11031, Taiwan.

2 Department of Neurology, School of Medicine, College of Medicine, Taipei Medical University, Taipei City 11031, Taiwan.

3 Division of Neurosurgery, Department of Surgery, Taipei Medical University-Shuang Ho Hospital, New Taipei City 23561, Taiwan.

4 Taipei Neuroscience Institute, Taipei Medical University, Taipei 11031, Taiwan.

5 International Ph.D. Program in Medicine, College of Medicine, Taipei Medical University, Taipei City 11031, Taiwan

6 Department of Medical Research & Education, Taipei Medical University-Shuang Ho Hospital, New Taipei City 23561, Taiwan

7 Continuing Education Program of Food Biotechnology Applications, College of Science and Engineering, National Taitung University, Taitung 95092, Taiwan

† These authors contributed equally to this work.

*Authors to whom correspondence should be addressed.

Dr. Heng-Wei Liu, PhD., Department of Medical Research and Education, Taipei Medical University - Shuang Ho Hospital, New Taipei City 23561, Taiwan. E-mail: henryway0404@hotmail.com

Prof. Chien-Min Lin MD., PhD, Division of Neurosurgery, Department of Surgery, Taipei Medical University-Shuang Ho Hospital, New Taipei City 23561, Taiwan. E-mail: m513092004@tmu.edu.tw

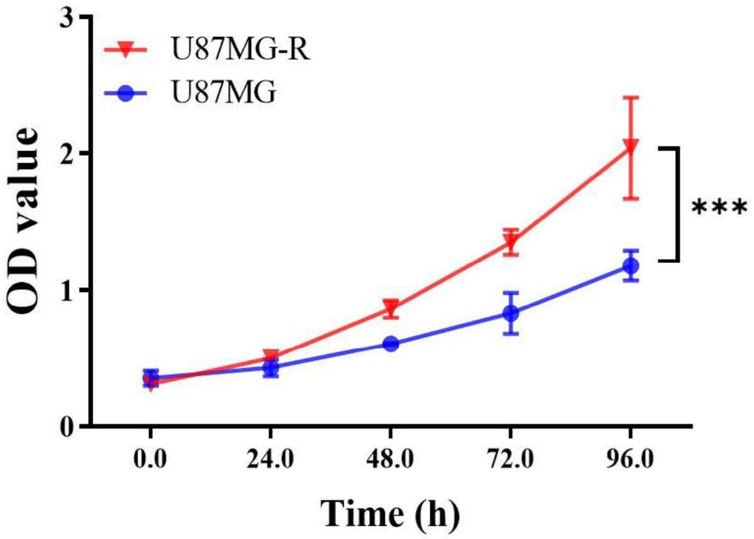

Figure S1. Increased cellular proliferation rate in U87MG-R than parental U87MG cells. Significance level: *** $p < 0.001$.

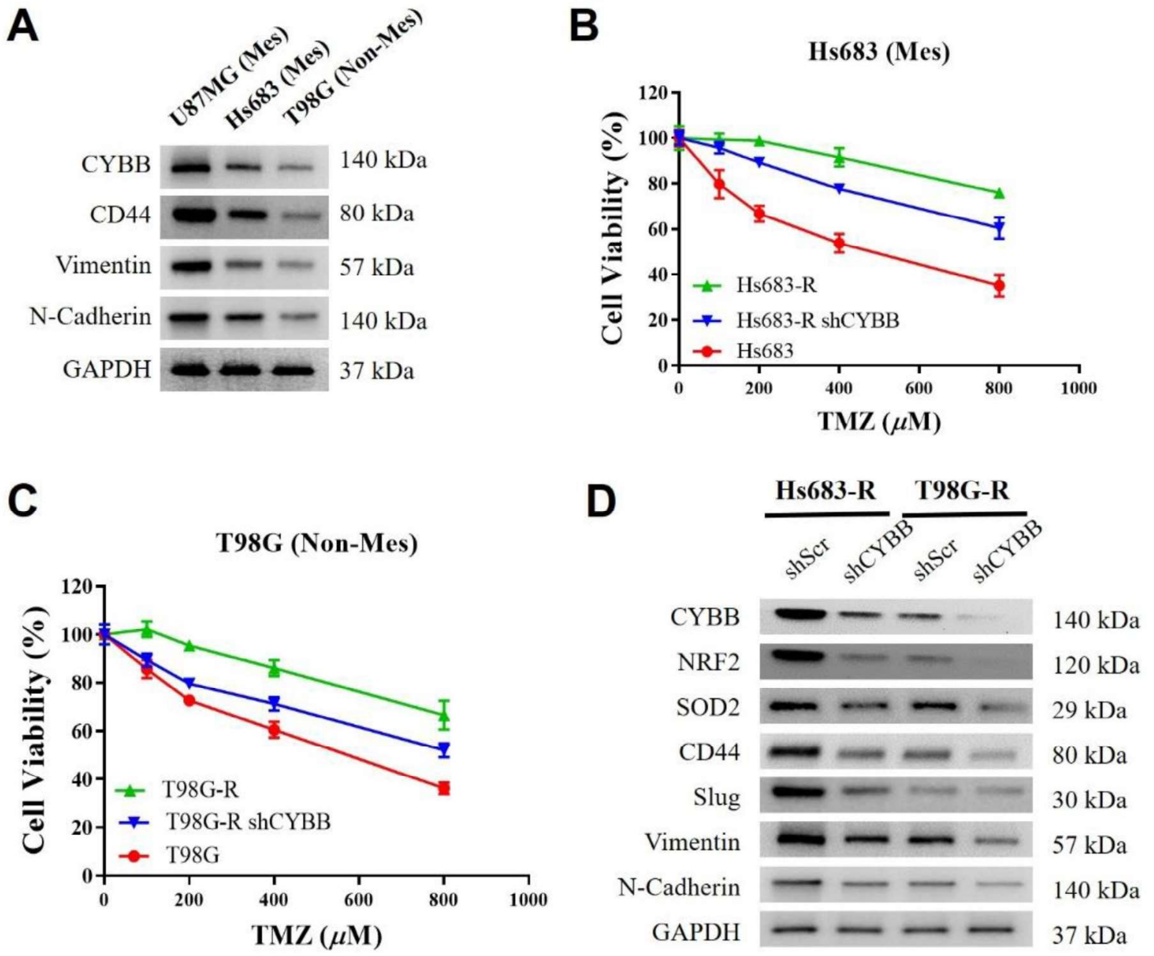

Figure S2. Role of CYBB in mesenchymal and non-mesenchymal GBM cell lines in the acquisition of TMZ resistance. (A) Western blotting indicated differential expression of CYBB and mesenchymal activation according markers (CD44, Vimentin, N-Cadherin) between presumed mesenchymal cells (U87MG, Hs683) and non-mesenchymal (T98G) GBM cells. The drug response curve showed relative differential sensitivity of TMZ treatment upon CYBB silencing in two chemoresistant GBM cell lines: Hs683 (B) and T98G (C). (D) Western blotting portrayed perturbation of NRF2/SOD2 axis activation and mesenchymal markers (CD44, Slug, Vimentin, N-Cadherin) between presumed Hs683-R and T98G-R chemoresistant GBM cells.
